# Numerical study on biomechanical analysis of human lumbar spine in daily activities

**DOI:** 10.7717/peerj.21851

**Published:** 2026-10-29

**Authors:** Bing Qin, Lu Zhou, Xiaoyu Zhang, Yuheng Liu, Zhiyu Qian, Qiaoqiao Zhu

**Affiliations:** 1 Department of Biomedical Engineering, Nanjing University of Aeronautics & Astronautics, Nanjing, China; 2 College of Information Engineering, Taizhou University, Taizhou, China

**Keywords:** Daily activities, Biomechanics, Musculoskeletal modeling, Lumbar spine, Motion capture

## Abstract

The aim of this study was to establish and apply a framework that combines musculoskeletal modeling with motion capture data to investigate the biomechanics of the human lumbar musculoskeletal system during daily activities. By combining musculoskeletal modeling with motion capture data and performing inverse dynamics analysis, we quantified the compressive and shear forces on the lumbar intervertebral disc (IVD), total muscle force, muscle activation, and ligament forces during six daily activities (slow walking, walking, fast walking, sweeping the floor, washing windows, and standing on one foot). Our results showed that the mechanical loading on the lumbar IVD, muscles and ligaments increased with the increase of walking speed, and the mechanical loading on most lumbar tissues during sweeping the floor and washing windows was greater than that during walking. The mechanical loading within each tissue (including the lumbar IVD, muscles and ligaments) showed consistent distribution characteristics across different activities. Our study showed that sweeping the floor, washing windows, and increased walking speed resulted in higher mechanical loading on the lumbar IVDs, muscles, and ligaments. Our findings are important for understanding the biomechanics of the lumbar spine during daily activities.

## Introduction

Low back pain is a major public health issue that affects a substantial proportion of the global population (Knezevic et al., 2021). It is reported that approximately 620 million people worldwide experience low back pain (GLBP Collaborators, 2023). Among 1,867 workers with a first episode of work-related, nonspecific low back injury, 17.2% developed recurrent work disability and 33.9% required recurrent medical care (Wasiak, Kim & Pransky, 2006). In daily life, the occurrence of low back pain is very common, and numerous studies have investigated how daily activities affect lower back health. Studies showed that physical activities such as housework were associated with increased risks of low back pain (Rosano et al., 2004; Ho et al., 2024). For example, 26.5% reported a history of lower back musculoskeletal injury in the past year, and 86.4% reported current low back pain during mopping tasks among 132 professional floor mopping cleaners (Naik & Khan, 2020). Other studies showed that prolonged flexed postures (no matter sitting or standing) were associated with elevated risks of low back pain (Khadour et al., 2025; Asfara, Paskarini & Wahyudiono, 2024). While some studies suggested that fast walking might reduce the incidence of low back pain (Maselli et al., 2020), others found that it increased loading on various tissues in the lumbar spine, potentially leading to adverse effects (Tojima, Osada & Torii, 2019). However, the specific effects of daily activities on lumbar spine health and the biomechanical mechanisms remain largely unclear.

Low back pain is related to the health of various tissues in the lumbar spine. Studies suggest that lumbar intervertebral disc (IVD) damage and degeneration are major contributors to low back pain (Wang et al., 2024), and spinal posture is also significantly associated with low back pain (Swain et al., 2020). The lumbar multifidus (MF) and transversus abdominis (TRA) were crucial for maintaining spinal stability (Qin et al., 2022; Ouyang et al., 2013). Atrophy of these muscles was related to low back pain (Qin et al., 2022; Colloca & Hinrichs, 2005). Spinal ligaments, including the interspinous (IS), supraspinous (SS), and flavum (FL) contributed to passive stability of the spine (Yoseph et al., 2025). Ligamentous dysfunction has also been associated with increased risk of low back pain. Muscle and ligament dysfunction can reduce spinal stability and increase the load on the lumbar IVD. Excessive mechanical loading on the lumbar IVD increases the risk of IVD degeneration (Vergroesen et al., 2015; Stokes & Iatridis, 2004). Therefore, quantifying the loading under daily activities is critical for understanding the biomechanics of low back pain. However, a quantitative study on the biomechanics of the lumbar spine during daily activities remains limited. Arshad et al. (2018) studied the forces of the lumbar spinal muscles during walking using musculoskeletal modeling, Zadoń et al. (2019) studied the loading on the lumbar IVD during sitting, standing, and lifting using musculoskeletal modeling. Most musculoskeletal modeling studies have focused on a single tissue or activity (*e.g.*, walking or standing), lacking comparisons of the mechanical responses of various spinal tissues (*e.g.*, IVD, muscle, ligament) across multiple daily activities within a unified modeling framework (Zadoń et al., 2019; Knapik et al., 2022; Lerchl et al., 2023; Favier et al., 2021).

Therefore, in this study, we aimed to establish and apply a framework that combines musculoskeletal modeling with motion capture data for inverse dynamics analysis. Using this framework, we quantified the compressive and shear forces acting on the lumbar IVDs, total muscle force, muscle activation, and ligament force during six daily activities, and compared the biomechanical responses of multiple lumbar tissues across activities. These quantitative biomechanical signals may help explain why certain activities (*e.g.*, fast walking, sweeping floor, washing window) are associated with higher low back pain risk. These findings contribute to a better understanding of the biomechanical mechanisms associated with activity-related low back pain.

## Methods

The biomechanics (including compressive and shear forces on the lumbar IVD, muscle forces and activation, ligament forces) of lumbar spine under six daily activities (including walking, slow walking, fast walking, sweeping the floor, washing windows, and standing on one foot) were quantitatively analyzed with a developed musculoskeletal model based on motion captured data.

### Motion capture

The motion capture dataset was obtained from the Carnegie Mellon University (CMU) Mocap Database (Lab, 2003). According to the database documentation, the motion capture was performed using 12 Vicon MX-40 cameras (Vicon Motion Systems Ltd., Oxford, UK) operating at 120 Hz in a 3 m × 8 m capture volume. Participants wore a black jumpsuit with 41 retro-reflective markers taped on (four markers on the head, seven markers on the trunk, 12 markers on the upper limbs, four markers on the pelvis, and 14 markers on the lower limbs). Camera images are triangulated to obtain three-dimensional marker coordinates, which are saved directly by the Vicon system as C3D files (Fig. 1A). The CMU database contains over 140 healthy adult subjects and 2,605 motion trials. Detailed subject demographics (age, gender, BMI) are not provided in the public release. One motion trial was selected for each of the six activities examined in this study: slow walking, walking, fast walking, sweeping the floor, washing windows, and standing on one foot. The trials were selected based on data completeness and motion quality. Specifically, the selected trials contained sufficiently complete motion records, clearly represented the target activities, and included complete movement cycles suitable for musculoskeletal modeling and inverse dynamics analysis. The selection was conducted at the trial level rather than according to the characteristics of a specific participant. These six activities were selected because they represented common daily motions with varying walking speeds (slow, normal, fast), trunk postures (flexion and torsion during sweeping and window washing), and balance demands (standing on one foot), thereby covering a representative range of physical demands encountered in daily life. All trials were processed using the same musculoskeletal modeling framework and consistent scaling and analysis procedures. The C3D files corresponding to six daily activities were extracted from the database and imported directly into the AnyBody Modeling System (AnyBody Technology, Version 7.4, Denmark) as input to drive the musculoskeletal model.

**Figure 1:**
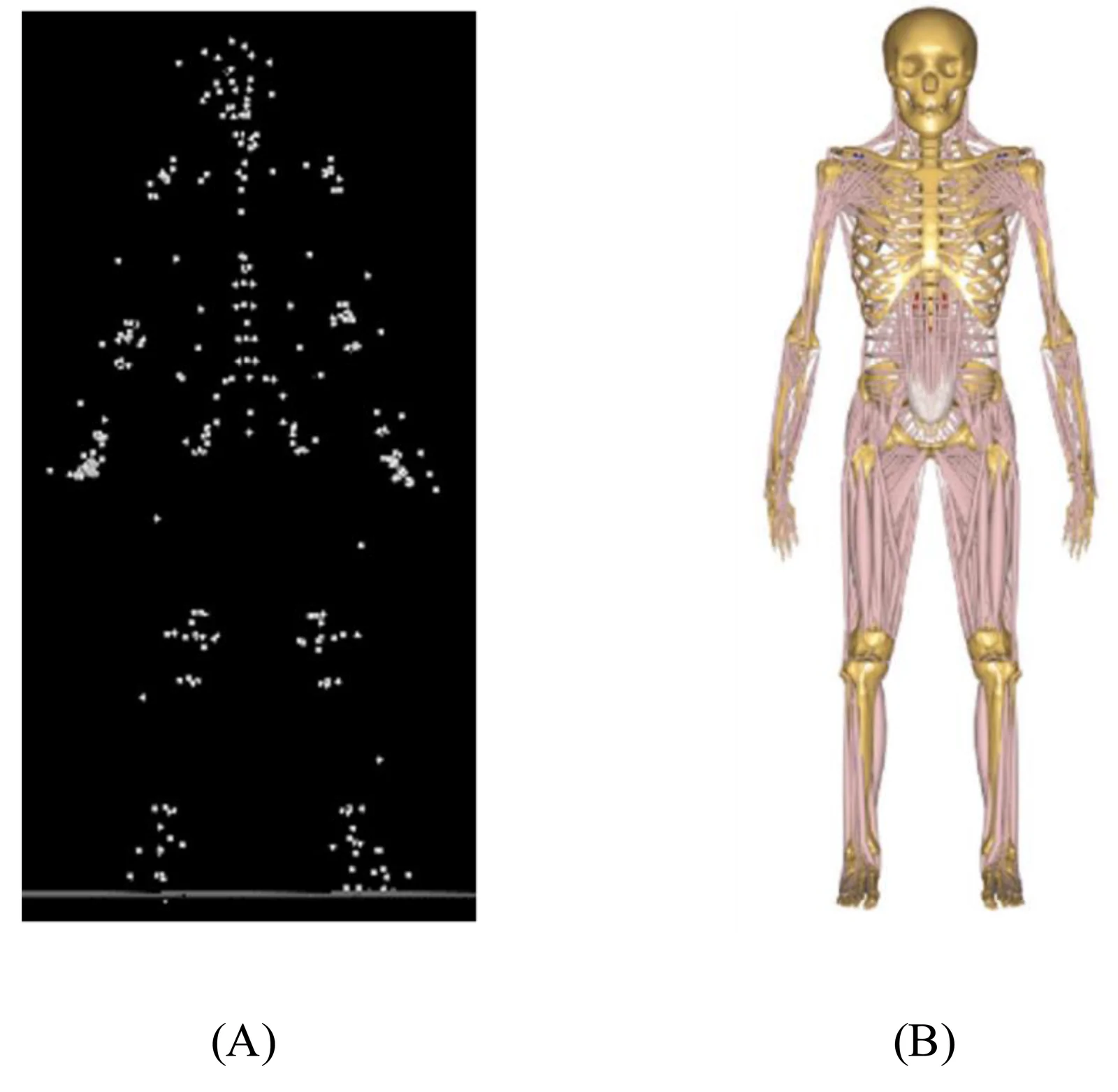
The markers positions in the 3D space acquired with motion capture software (A) and subsequently imported to set the AnyBody mannequin kinematics (B).

### Musculoskeletal model

A full body musculoskeletal model was developed using the AnyBody Modeling System. Specifically, within the spine, five vertebral bones (including vertebra L1-L5) were modeled as rigid bodies; five IVDs (including L1L2, L2L3, L3L4, L4L5, and L5S1) were modeled as spherical joints with three degrees of freedom; the muscles (including lumbar MF, erector spinae (ES), psoas major (PM), quadratus lumborum (QL), obliquus externus (OE), obliquus internus (OI), semispinalis (SR), thoracic multifidus (TMF), rectus abdominis (RA), and TRA) were modeled as follow: the maximum strength of the lumbar muscles was defined as the multiplication of the functional cross-sectional area (FCSA) of the muscle and a strength coefficient. Muscle recruitment was solved using the ‘minimize muscle activation squared’ criterion, which was the default optimization strategy in AnyBody Modeling System. This criterion assumed that the central nervous system distributes muscle forces so as to minimize the sum of squared muscle activations, subject to equilibrium and force constraints. Muscle activation *a* is defined as *a*=*F*_*m*_*/F*_0_, where *F*_*m*_ is the computed muscle force and *F*_0_ is the maximum isometric force parameter. The maximum isometric force parameter *F*_0_ of each muscle is proportional to its functional cross-sectional area (FCSA). Activation values reported in this study were expressed as a percentage of the maximum isometric force capacity of each muscle. The geometry of the lumbar muscles (origin, insertion, and muscle path) and the attachment points of the lumbar ligaments were based on the AnyBody Modeling System default lumbar spine model. This model was derived from anatomical data and cadaveric studies. Muscle paths were represented as line segments using via-points or wrapping surfaces as appropriate. The mechanical behaviors of ligaments (including anterior longitudinal ligament (ALL), posterior longitudinal ligament (PLL), IS, SS, FL, and intertransverse (IT)) were characterized using nonlinear elastic models (Fig. 1B), and their insertion coordinates followed standard anatomical descriptions. This model has been validated against *in vivo* IVD pressure data under 12 different daily activities. More details could be found in our previous publications (Qin et al., 2022). The full body musculoskeletal model was combined with the selected motion capture data, and the mechanical responses of the lumbar spine during the six activities were quantified through inverse dynamics analysis.

The musculoskeletal model used in this study has been validated in our previous work (Qin et al., 2022). Specifically, the model’s predicted compressive force on the IVD was compared against *in vivo* measurements under 12 different daily activities. The prediction errors were within 10%, showing that the model can accurately estimate the compressive forces on the lumbar IVDs under daily posture.

## Results

### Compressive force on the lumbar IVD

The compressive force on the lumbar IVD varied considerably across different activities. Specifically, the average compressive forces on the lumbar IVD were 809 N, 1220 N, 1396 N, 1425 N, 2192 N and 2211 N during slow walking, walking, fast walking, standing on one foot, washing windows, and sweeping the floor, respectively (Fig. 2A) (Table 1). Across most activities, the compressive force distribution on the lumbar spine exhibited a descending pattern. Specifically, the L1L2 IVD experienced the highest compressive force, followed by L2L3 IVD, L3L4 IVD and L4L5 IVD, with the L5S1 IVD consistently exhibiting the lowest compressive force (Fig. 2B).

**Figure 2:**
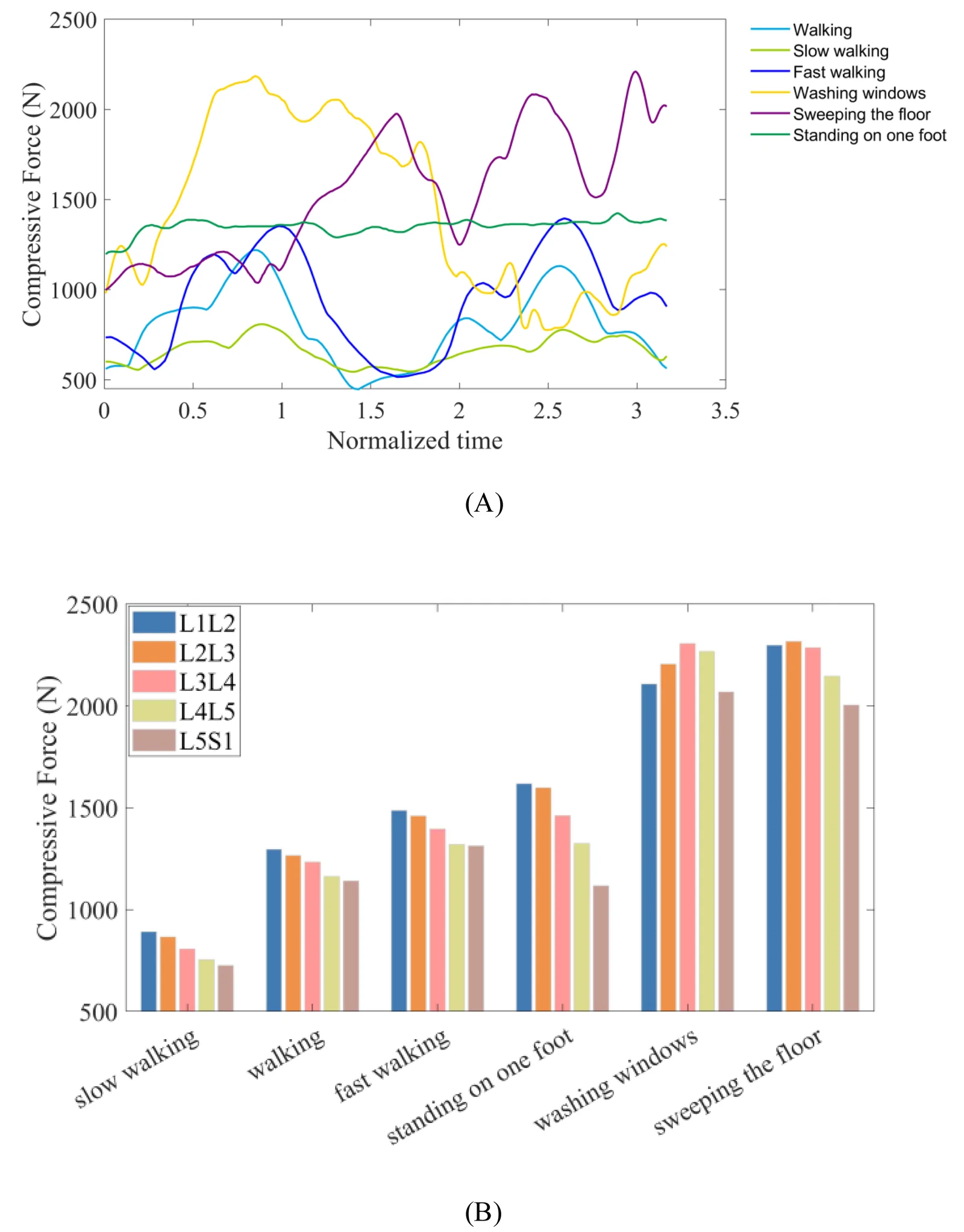
Comparison of compressive forces on the lumbar IVD during the six daily activities. (A) the average compressive forces among all lumbar IVDs, (B) the maximum compressive forces on each lumbar IVD. The duration of each activity was normalized to the duration of the slow walking cycle.

### Shear force on the lumbar IVD

The shear force on the lumbar IVD varied considerably across different activities. Specifically, the average shear force on the lumbar IVD were 160 N, 240 N, 257 N, 304 N, 578 N, 457 N during slow walking, walking, fast walking, standing on one foot, washing windows, and sweeping the floor, respectively (Fig. 3A) (Table 1). Across most activities, the shear force distribution on the lumbar spine exhibited a descending pattern. Specifically, the L5S1 IVD experienced the highest shear force, followed by L1L2 IVD, L2L3 IVD and L4L5 IVD, with the L3L4 IVD consistently exhibiting the lowest shear force (Fig. 3B).

### Muscle activation & the total muscle force

The total muscle force (defined as the sum forces of all ten muscle groups related to the lumbar region) and the activation of different muscle groups show differences in daily activities. The muscle activations were 7%, 12%, 15%, 6%, 16%, and 18%, and the total muscle forces were 1178 N, 1878 N, 2254 N, 1338 N, 4041 N, and 3306 N during slow walking, walking, fast walking, standing on one foot, washing windows, and sweeping the floor, respectively (Figs. 4A and 5A) (Table 1). The TRA showed the greatest activation and the ES showed the greatest force among all activities. Specifically, as walking speed increases, there is a corresponding increase in the total muscle force and the activation of most muscles (Figs. 4 and 5).

**Table 1:** Summary of average mechanical responses during the six daily activities. Values for compressive force, shear force, muscle activation, and ligament force were presented as mean ± standard deviation (SD). Compressive and shear forces are averaged across all five lumbar IVDs (L1L2 to L5S1). Muscle activation is averaged across all lumbar muscle groups. Total muscle force is the sum of all ten lumbar muscle groups. Ligament force is averaged across all six lumbar ligaments.

	Slow walking	Walking	Fast walking	Standing on one foot	Washing windows	Sweeping the floor
Compressive force (N)	809 ± 70	1,220 ± 66	1,396 ± 80	1,425 ± 208	2,192 ± 102	2,211 ± 134
Shear force (N)	160 ± 95	240 ± 127	257 ± 140	304 ± 209	578 ± 284	457 ± 271
Muscle activation (%)	7 ± 4	12 ± 6	15 ± 8	6 ± 3	16 ± 11	18 ± 11
Total muscle force (N)	1,178	1,878	2,254	1,338	4,041	3,306
Ligament force (N)	225 ± 144	217 ± 139	203 ± 129	637 ± 327	710 ± 366	592 ± 307

**Figure 3:**
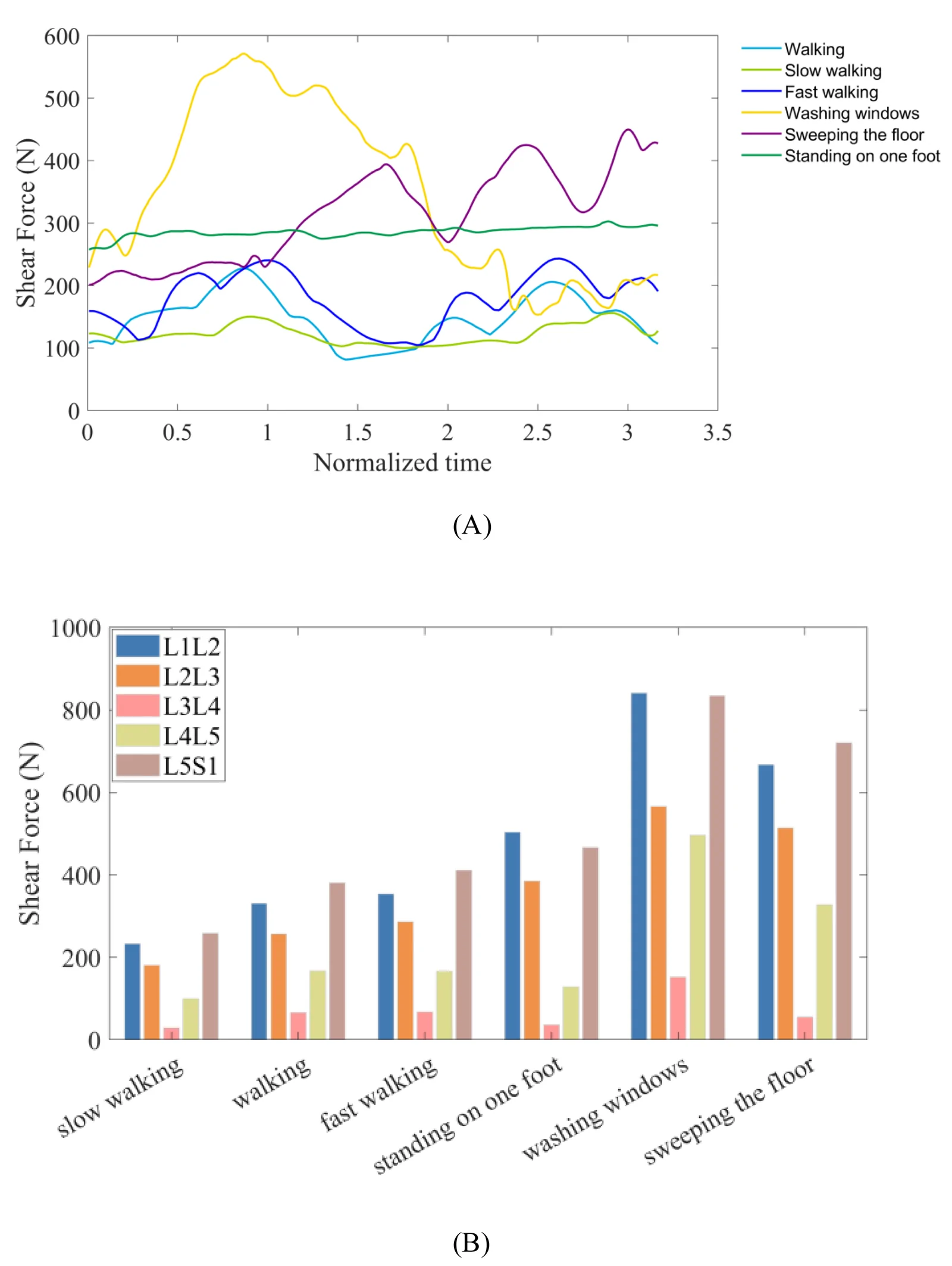
Comparison of shear forces on the lumbar IVD during the six daily activities. (A) the average shear forces among all lumbar IVDs, (B) the maximum shear forces on each lumbar IVD. The duration of each activity was normalized to the duration of the slow walking cycle.

**Figure 4:**
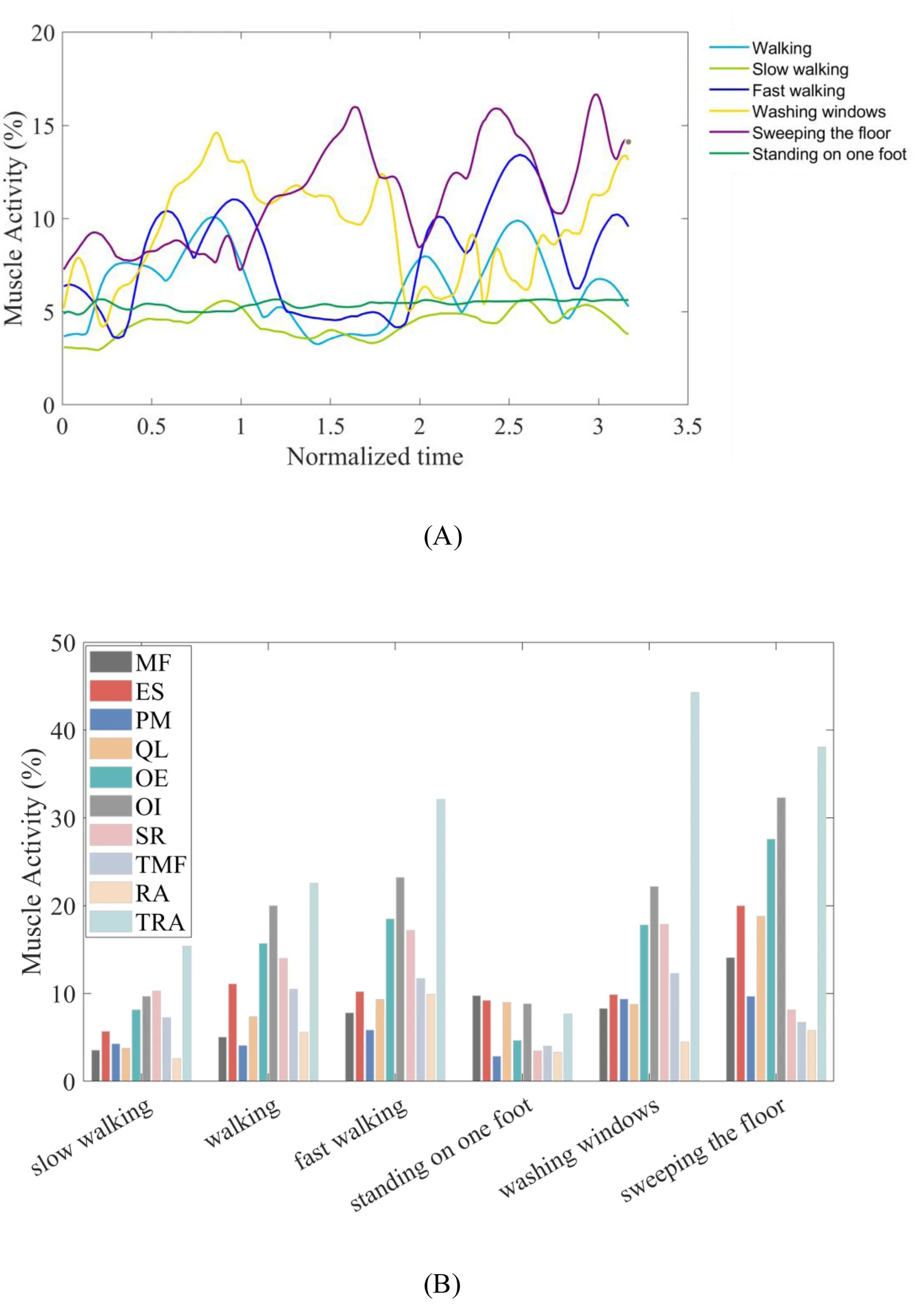
Comparison of muscle activation during the six daily activities. (A) the average activation among all muscle groups, (B) the maximum activation of each muscle group. The duration of each activity was normalized to the duration of the slow walking cycle. Muscle activation is expressed as a percentage of the maximum isometric force capacity of each muscle. (MF, multifidus; ES, erector spinae; PM, psoas major; QL, quadratus lumborum; OE, obliquus externus; OI, obliquus internus; SR, semispinalis; TMF, thoracic multifidus; RA, rectus abdominis; TRA, transversus abdominis).

**Figure 5:**
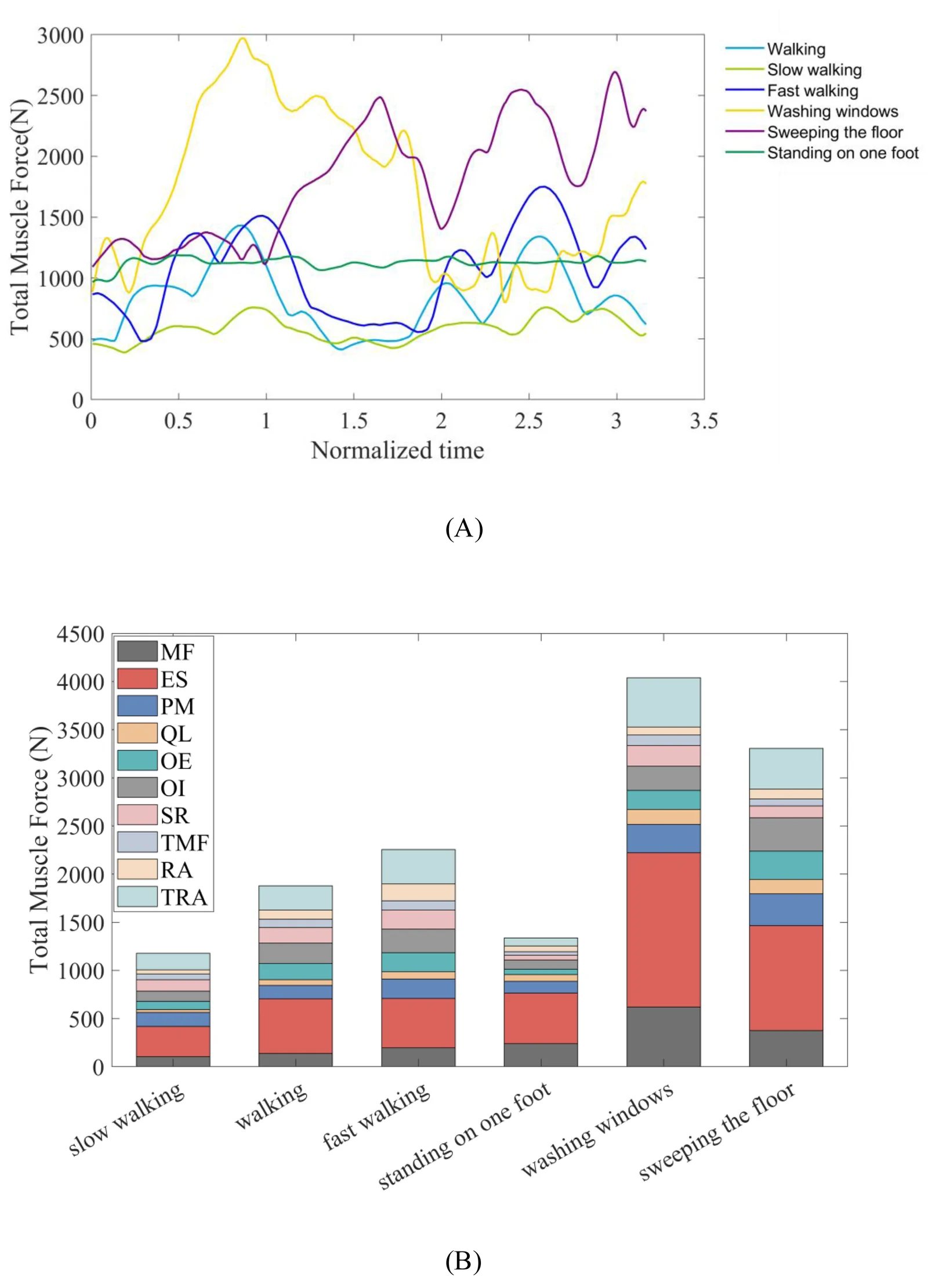
Comparison of total muscle force during the six daily activities. (A) the total muscle force across all muscle groups, (B) the maximum total muscle force of each muscle group. The duration of each activity was normalized to the duration of the slow walking cycle. (MF, multifidus; ES, erector spinae; PM, psoas major; QL, quadratus lumborum; OE, obliquus externus; OI, obliquus internus; SR, semispinalis; TMF, thoracic multifidus; RA, rectus abdominis; TRA, transversus abdominis).

### Ligament force

The forces in the ligaments show differences in daily activities. The ligament forces were 225 N, 217 N, 203 N, 637 N, 710 N, 592 N during slow walking, walking, fast walking, standing on one foot, washing windows, and sweeping the floor, respectively (Fig. 6A) (Table 1). The FL showed the greatest force among all activities. Specifically, the ligament forces during standing on one foot, washing windows, and sweeping the floor were higher than those during walking activities (slow walking, walking and fast walking) (Fig. 6B).

**Figure 6:**
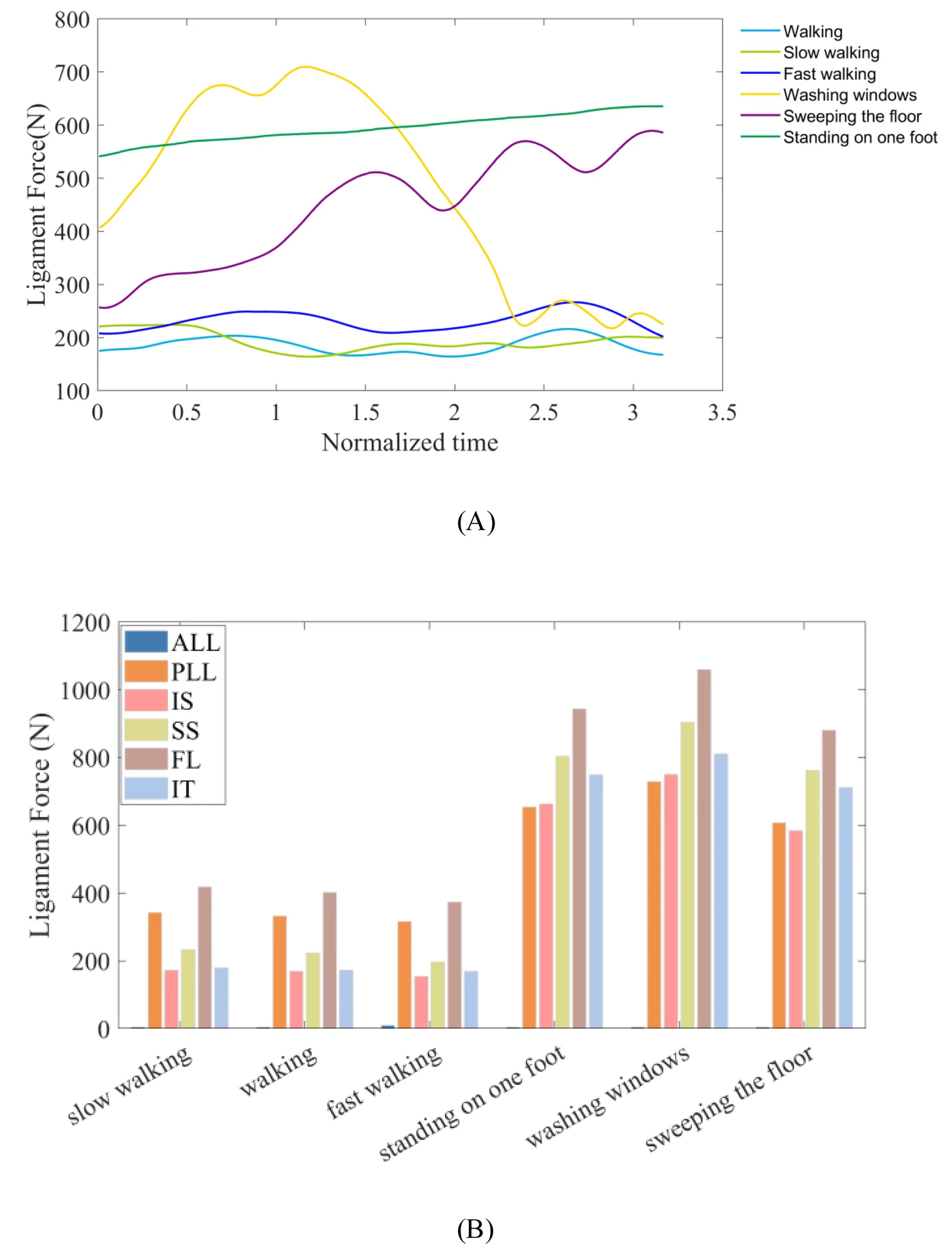
Comparison of ligament force during the six daily activities. (A) the average force among all ligaments in the lumbar spine, (B) the maximum force of each ligament. The duration of each activity was normalized to the duration of the slow walking cycle. (ALL, anterior longitudinal ligament; PLL, posterior longitudinal ligament; IS, interspinous; SS, supraspinous; FL, flavum; IT, intertransverse).

## Discussion

The lumbar spine biomechanics under various daily activities (including walking, slow walking, fast walking, sweeping the floor, washing windows, and standing on one foot) were quantitatively analyzed using musculoskeletal modeling based on the motion captured data. We found that the mechanical loading on the lumbar IVD, lumbar muscles and ligaments increased with the increase of walking speed, and the mechanical loading on most tissues during sweeping the floor and washing windows was greater than that during walking activities. We also found that the mechanical loading within each tissue (including the lumbar IVD, muscles and ligaments) showed consistent distribution characteristics across different activities. To the best of our knowledge, this is the first study that quantitatively analyzed the mechanical loading on the lumbar spine under various daily activities.

Our results showed that the compressive and shear forces on the lumbar IVD increased during walking compared with those during slow walking, these increases were more remarkable during fast walking, which was consistent with previous research results (Cheng et al., 1998; Nahhas Rodacki et al., 2024). During faster walking, the spine flexes forward to maintain body balance, which is associated with greater mechanical loading on the lumbar IVD (Carson, Aslan & Ortega, 2024). Moreover, our results showed that the mechanical loading on the lumbar IVD increased during sweeping the floor and washing windows compared to those during walking. This is because the spinal flexion and torsion during these two activities would lead to an increase of the compressive and shear forces on the lumbar IVD (Qin et al., 2022; Wilke et al., 2001). Studies have reported the association between spinal flexion as well as torsion and low back pain (Swain et al., 2020), and that an increase of compressive force on the lumbar IVD would increase the risk of lumbar IVD damage and/or degeneration (Stokes & Iatridis, 2004). Our results showed that the compressive force on all lumbar IVD were greater than 2000 N during sweeping the floor and washing windows, while studies have reported that compressive forces above 2000 N may initiate damage at the posterior regions in the annulus fibrosis (Gao, Zhu & Gu, 2017), indicating a risk of IVD damage and/or degeneration during these activities. It was suggested that the mechanical loading on the lumbar IVD during sweeping the floor and washing windows increased, leading to a higher incidence of low back pain, which was consistent with the report that professional cleaners have a high incidence of low back pain (Naik & Khan, 2020).

Our results showed that the total muscle force and most muscle activation increased with the increase of walking speed. We also found that the total muscle force and most muscle activation during sweeping the floor and washing windows were greater than those during various walking activities. The shear force on the lumbar IVD increased with the increase in walking speed, and the shear force on the lumbar IVD during sweeping the floor and washing windows was greater than those during walking activities. Excessive shear force on the lumbar IVD might compromise spinal stability (Gallagher & Marras, 2012), and the muscles in the lumbar spine played a crucial role in maintaining the stability of the spine (Panjabi et al., 1989; Choi & Kim, 2012; Bojairami & Driscoll, 2022). Therefore, greater muscle strength was needed for the spine to maintain its stability during fast walking, sweeping the floor and washing windows. Furthermore, the activation of the MF and TRA showed markedly higher values during floor sweeping and window washing compared with walking. These activities involved trunk flexion and rotation, which challenge spinal stability and required greater involvement of stabilizing muscles. The study by Müller et al. (2021) showed that the loading on muscles increased when the range of motion of the lumbar increased, especially those muscles related to postural stability. Studies have reported that the cross-sectional area of the MF and TRA significantly decreased, the fatty infiltration increased in patients with low back pain (Kalli et al., 2026; Rahmani, 2013). The stabilizing capacity of these muscles was weakened and the normal physiological function of the core muscle group was affected when they atrophied, which may lead to abnormal loading and low back pain. This can help explain why people who frequently experienced the floor sweeping and window washing were more prone to low back pain developing.

The ligament force showed no significant difference among various walking activities, and the ligament forces during standing on one foot, sweeping the floor and washing windows were greater than those during walking activities. The ligaments in the lumbar spine played a crucial role in maintaining the stability of the spine (Yingling & McGill, 1999; Widmer et al., 2020). Our results showed that ligament force significantly increased during standing on one foot, sweeping the floor and washing windows compared to those during walking activities. This might be explained by the need to maintain body balance during single-leg stance, greater ligament force was required to counteract lateral instability of the spine. The flexion, extension, and rotation of the lumbar spine during sweeping the floor and washing windows led an increase in ligament force to mitigate the excessive deformation of the spine and maintain the stability of the spine (Zarei et al., 2018; Zander, Rohlmann & Bergmann, 2004). Prolonged exposure to high mechanical loading could contribute to ligament strain. Our results indicated that ligament forces were notably higher during standing on one foot, sweeping, and window washing, which might increase the risk of fatigue injury in lumbar spinal ligaments.

A study has reported the activation of the MF and the ES increased when the walking speed increased using electromyography (EMG), which was consistent with our results (Bryndal et al., 2025). Studies have reported that the compressive force on the lumbar IVD increased significantly under the flexion and bending loading using finite element analysis, which was consistent with our results (Kuo et al., 2010; Schmidt et al., 2007). A study has reported that the compressive and shear forces on the lumbar IVD, the forces of major trunk muscles increased significantly during fast walking compared with normal walking using musculoskeletal modeling (Jamshidian et al., 2025). A study also has reported that the compressive forces on the lumbar IVD increased under lifting (Yoseph et al., 2025). These findings were consistent with our results that the loading on the IVD and muscle increased under fast walking, lumbar flexion and bending. However, none of these previous studies have quantified the effect of various daily activities on the loadings on IVDs, muscles, and ligaments within a unified framework. This study quantified the biomechanical responses of IVDs, muscles, and ligaments during six daily activities, which may contribute to a better understanding of the biomechanical mechanisms of low back pain in daily activities.

This study has some limitations. First, the lumbar IVD in the musculoskeletal model was simplified as a rigid spherical joint, the swelling and deformation of the lumbar IVD under compressive loading was not considered. This simplification was adopted because the aim of this study was to quantify the total mechanical loads (compressive and shear forces) on the IVDs, rather than the internal stress or strain distribution within the IVD. Our validation showed that the deviation between the compressive force on the IVD predicted by this simplified model and the *in vivo* measurement values was within 10%. Therefore, the simplification did not substantially affect the primary outcomes of this study. Moreover, including IVD swelling and deformation would increase computational cost by several orders of magnitude, making dynamic simulations of multiple activities infeasible. Future work may adopt finite element models to analyze stresses within the IVD. Second, the subjects in the motion capture dataset were healthy and had no spinal diseases, and the biomechanical properties of each tissue in the musculoskeletal model were also determined based on healthy people. The effects of spinal degeneration (such as lumbar IVD degeneration and muscle atrophy, which were frequently observed in individuals who perform household chores) on its biomechanics were not considered in this study. Further research is needed to fully understand the influence of spinal degeneration on spinal biomechanics.

In conclusion, we established and applied a framework that combines motion capture data with musculoskeletal modeling to investigate the biomechanics of the human lumbar musculoskeletal system during six daily activities. Using inverse dynamics analysis, we quantified the responses of the lumbar IVD, muscles, and ligaments and compared their loading patterns across different activities. We found that the mechanical loading on the lumbar IVD, muscles and ligaments increased with the increase of walking speed, and the mechanical loading on most spinal tissues during sweeping the floor and washing windows was greater than that during various walking activities. These findings provide better understanding of how daily activities affect the biomechanics of the human lumbar spine, which is important for understanding the effect of daily activities on the health of human lumbar musculoskeletal system.

## Additional Information and Declarations

### Data Availability

The following information was supplied regarding data availability: The data is available at Zenodo: Qin, B. (2026). data for lumbar spine analysis during six daily activities [Dataset]. Zenodo. https://doi.org/10.5281/zenodo.22745355
The Motion Capture Data is available at: https://mocap.cs.cmu.edu/.
